# The Beach Bioreactor: Unraveling the Anomaly of Intensive Remineralization above a Deep Oxycline

**DOI:** 10.1021/acs.est.5c05752

**Published:** 2025-09-12

**Authors:** Felix Auer, Janek Greskowiak, Rena Meyer, Anja Reckhardt, Moritz Holtappels

**Affiliations:** † Alfred Wegener Institute Helmholtz Center for Polar and Marine Research, Am Handelshafen 12, Bremerhaven 27570, Germany; ‡ Institute of Biology and Environmental Sciences, 11233 Carl von Ossietzky Universität Oldenburg, Ammerländer Heerstraße 11, Oldenburg 26129, Germany; § Institute for Chemistry and Biology of the Marine Environment (ICBM), Carl von Ossietzky Universität Oldenburg, Carl-von-Ossietzky-Straße 9-11, Oldenburg 26129, Germany

**Keywords:** beach aquifer, subterranean estuary, oxygen consumption, organic carbon turnover, reactive transport model, biogeochemistry

## Abstract

High-energy beaches receive high inputs of organic matter from seawater infiltration, fueling intensive oxygen (O_2_) consumption rates in the upper sand layer, which depend on seasonal variations in temperature and organic matter supply and can exceed 10 times the average subtidal rates during the growing season. Despite the intensive rates, recent studies have found deep O_2_ penetration underneath, extending down several meters. To investigate this anomaly, we applied a reactive transport model to simulate O_2_ supply and consumption for winter and summer conditions. Well-known conditions at a high-energy beach on Spiekeroog Island, Germany, were used to define topography, hydraulic conductivity, tide, and wave amplitudes, and the model was built using measured O_2_ distribution and O_2_ consumption rates. The tide-resolving model was capable of simulating the periodic tidal desaturation of the surface layer. We found that the aeration with atmospheric O_2_ during desaturation is a significant O_2_ source, contributing up to 30–60% of total O_2_ consumption. Meanwhile, seawater O_2_ quickly bypasses the upper reactive layer, extending the oxycline to depths of 11–16 m in the summer and winter, respectively. This mechanism mitigates the seasonal imprint into deeper layers yet promotes intensive aerobic OC remineralization of up to 0.7 gC m^–2^ d^–1^.

## Graphical abstract

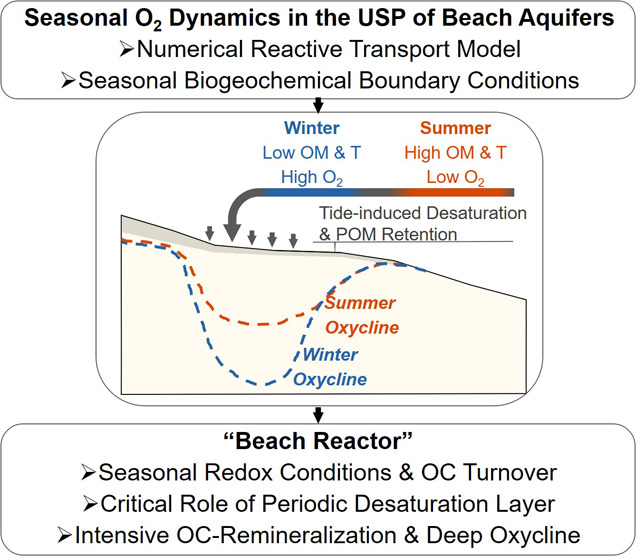

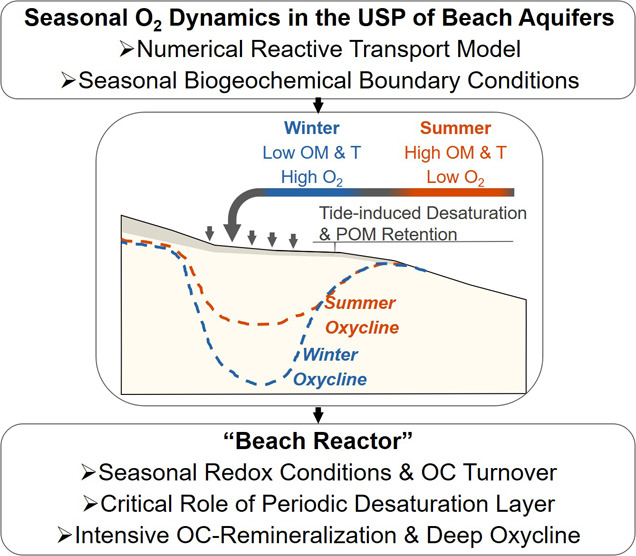

## 1 Introduction

Coastal aquifers are considered dynamic biogeochemical reactors at the land–ocean interface,^1–3^ where substances undergo significant modification before discharging to the ocean via submarine groundwater discharge.^4,5^ The permeable sands of beach interiors allow rapid advective transport of dissolved reactants along subsurface flow paths,^6,7^ feeding microbial communities attached to the grain surfaces.^8,9^ Tides and waves drive seawater infiltration and form a subsurface seawater recirculation cell, termed the upper saline plume (USP, Figure 1a),^10^ further introducing reactants such as oxygen (O_2_) and fresh organic matter (OM) that stimulate biogeochemical reactions in the otherwise organic carbon (OC)-poor beach sands.^4,11,12^ Surface and deep layers are tightly coupled by rapid advective transport of dissolved reactants, so that the physical and chemical boundary conditions at the upper infiltration zone affect the microbial turnover in the deeper beach bioreactor.^13,14^

**Figure 1:**
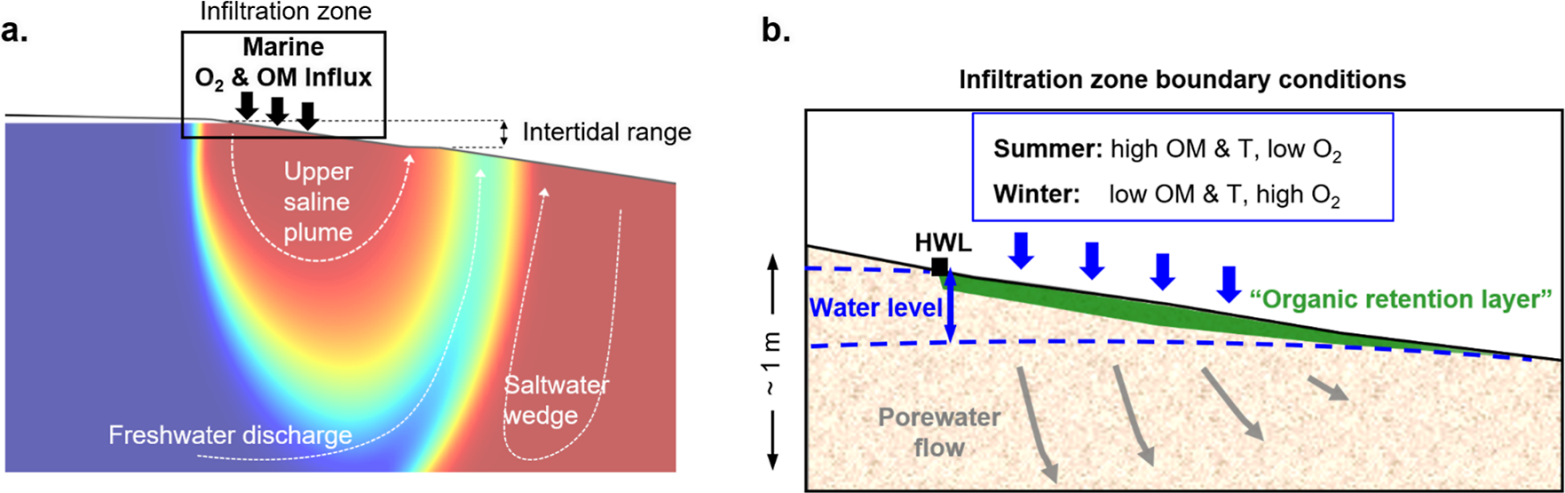
(a) General flow of saltwater (red) and freshwater (blue) in the beach aquifer. The black box marks the seawater infiltration zone as the boundary where fresh biogeochemical reactants (O_2_ and OM) enter the reactor. (b) Processes and boundary conditions in the seawater infiltration zone. Infiltrated seawater (blue arrows) delivers seasonally varying amounts of reactants, with particulate organic matter (POM) retained in the shallow organic retention layer (green) and dissolved phases transported further with porewater flow. The aquifer water level fluctuates periodically (dashed blue line), creating a zone of periodic desaturation in the upper sediment, where reaeration with atmospheric O_2_ occurs.

The shallow seawater infiltration zone receives large amounts of O_2_, promoting rapid aerobic degradation of OM.^11^ Due to the filtration effect of sands, particulate OM (POM) accumulates in the upper sediments, creating steep vertical gradients in O_2_ consumption rates with very high rates at the surface declining to low rates at depth.^15,16^ Especially in summer, increased seawater POM concentrations and elevated temperatures drive intensive OC remineralization in the infiltration zone.^16^ Despite the high O_2_ demand, recent field observations at a high-energy beach reported deep O_2_ penetration, extending to several meters depth.^17^ This anomaly raises questions about the mechanisms regulating O_2_ transport and redox zonation of the O_2_ under varying seasonal and hydrodynamic forcing.

Here, high-energy beaches are defined using a 2-dimensional tide-wave space bounded by macro-tidal ranges (>3.5 m) and high mean significant wave heights (>1.5 m), as recently described by Massmann et al.^18^ At high-energy beaches, seawater infiltration rates are high, and combined with morphodynamic variability, this generates highly dynamic biogeochemical conditions tens of meters below the surface.^14,19,20^ Seasonal changes in seawater composition, particularly OM supply, further regulate redox zonation and element fluxes via strong bentho–pelagic coupling.^12,21,22^ Yet, it remains unclear how the strong seasonal variability of biogeochemical inputs and aerobic remineralization in the upper beach layer affects redox zonation in the deep bioreactor. In addition to the seasonal supply of OM and O_2_ by seawater infiltration, periodic tidal desaturation of the upper sand layer allows for intermittent influx of atmospheric O_2_,^23–25^ a phenomenon that is largely unexplored with respect to its impact on subsurface biogeochemistry. A better understanding of these processes is essential to assessing the role of the beach aquifer in OC remineralization and to quantifying the flux of nutrients and trace elements.

We hypothesize that the strong seasonal variation in O_2_ consumption in the upper layer regulates O_2_ supply to the deeper USP, leading to a strong seasonal shift of the oxycline. However, this oxycline shift is likely affected by other processes. For example, a seasonal change in beach slope affects seawater volume flow as a whole or by the tide-dependent aeration of the periodically desaturated surface layer with atmospheric O_2_, which affects the O_2_ budget in particular. Especially, the latter would increase the importance of the upper infiltration zone as a critical chemical boundary layer (Figure 1b), influencing the entire beach reactor and contributing significantly to overall aerobic OM turnover.

Here, we exploit recently published high-resolution O_2_ consumption and O_2_ concentration measurements of a well-studied beach section as input for a model study. Our numerical reactive transport model uses O_2_ as a reactive tracer to investigate how seasonal and tidal variability in the upper infiltration zone determines the location and variability of the oxycline and thus the aerobic carbon mineralization rates within the entire USP. The gained process understanding of the beach bioreactor is essential to assess the contribution of high-energy beaches to the coastal element cycling.

## 2 Numerical Reactive Transport Model

A two-dimensional cross-sectional numerical model was developed to simulate the tidal and wave-driven groundwater flow and reactive O_2_ transport in the upper saline plume (USP, Figure 1a) along a cross-shore beach transect of the unconfined beach aquifer at Spiekeroog Beach, Germany. For details on the location of the study site and infrastructure, we refer to Massmann et al.^19^ All model development and simulations were performed using the finite element analysis software COMSOL Multiphysics v. 6.1.^26^ The Module “Subsurface Flow” was used to simulate Darcy flow with the “Richards Equation” interface for variably saturated media. The Module “Transport of Diluted Species in Porous Media” was used to simulate reactive O_2_ transport. The boundary conditions and the domain parameters were adopted from field measurements.^16,17,27^ This includes measurements of tidal amplitudes, waves, and topography as well as high-resolution measurements of sedimentary O_2_ consumption rates, porewater O_2_ concentration, and sediment grain size distribution in the upper layer of 4 stations (IT 1–4) along a transect of the infiltration zone (Figure 2a). Simulations were conducted in two stages, by first modeling time-resolved groundwater flow during a tidal cycle and then periodically coupling the transient flow with the reactive model. In the following, we present the implementations of the key boundary conditions and parameters. Additional details on the two model stages and boundary conditions are described in Supporting Information Sections 1.1 and 1.2. A summary of the parameters used in the transport and reactive models is given in Tables S1 and S2.

**Figure 2:**
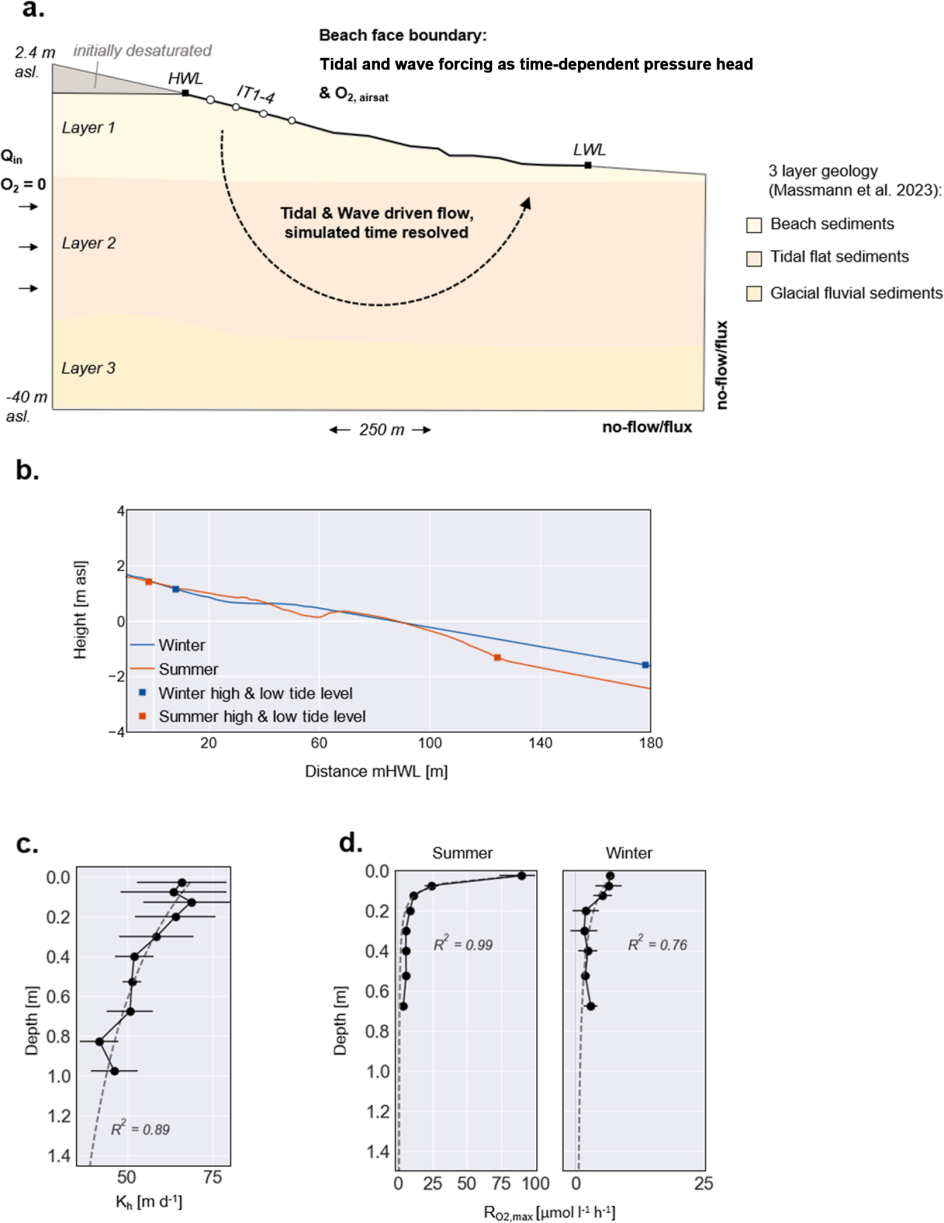
(a) Schematic setup of the numerical model including the shape of the 3-layer geology as found in geological investigations. (b) Intertidal beach profiles for summer (measured in June 2022) and winter (measured in December 2022) including respective high and low tide levels. (c) Hydraulic conductivity estimates in the upper layer based on grain size d10 of the beach sediments (values from June 2022 as a representative case). The dashed line shows the fit function that was used to construct the hydraulic conductivity for the upper geological beach layer. (d) Profiles of measured O_2_ consumption rates in the upper 70 cm for the summer and winter case. The dashed line shows the fit function that was used to implement O_2_ consumption in the model domain.

The model domain extends 250 m horizontally from south to north, starting at 50 m inland of the mean high-water line (mHWL, at 1.37 m above average sea level [asl.]), and encompasses the intertidal beach aquifer (Figure 2a). The measured intertidal beach topography was incorporated into the model and extended both landward and seaward using the mean beach slope from the measurements (Figure 2b). To cover seasonal variations, we simulated two scenarios based on data collected during a summer and a winter field campaign. June 2022 was selected to represent summer conditions with a pronounced reactive layer, while December 2022 was used to capture winter conditions (Figure 2). The summer profile represents a more reflective beach with a slightly steeper slope (mean slope of 1.9% in summer vs 1.7% in winter), typical for summer months.^28^ Therefore, the intertidal zone is about 36 m larger in the winter scenario. Vertically, the model extends from 2.4 m above to 40 m below average sea level (asl.) where the base of the aquifer is situated according to previous studies.^14,29^ The grid had 90 layers and 300 columns. A fine vertical discretization of 0.05 m was implemented in the shallow subsurface. Vertical discretization increases with depth to a maximum of 1.7 m at the domain bottom, and the model was tested for grid independence. The horizontal discretization is 0.8 m.

At initial conditions, the model domain is fully saturated below the respective height of the high water line (see also Table S1). Porewater transport in the model is driven by diurnal tides and wave setup at the beach-face boundary and by freshwater discharge *Q*_in_ of 0.51 m^3^ m^–1^ d^–1^ ^30^ at the land–ward boundary (Figure 2a). Tidal forcing is applied on the beach boundary as a time-dependent head boundary condition (first-type boundary) when water levels exceed topographic height at location x (see also eq S1). Otherwise, a seepage face is present at this boundary. The tidal amplitude of 1.37 m represents the average forcing at the respective north-facing beach.^29^ The mean significant wave height of 0.59 m was taken from wave data at Nordergründe ∼30 km to the East of Spiekeroog beach provided by the Federal Maritime and Hydrographic Agency.^27^ As the primary model focus is on O_2_ transport in the USP driven by tidal and wave forces, a salinity gradient is not included in the model and density-dependent flow was not accounted for to limit computational efforts. While this is a simplified approach, density-dependent flow is not expected to significantly impact the flow regime in the oxic part of the USP. The modeled pore water transport depends strongly on the hydraulic conductivity of the beach, which is often subject to large uncertainties. In this study, the hydraulic conductivity of the model was derived from geological investigations at the field site during the drilling of three multilevel wells down to 24 m depth. Three different sand layers were identified in the intertidal beach aquifer (Figure 2a).^19,31^ Tidal flat sediments of heterogeneous medium to fine sands with peat and clay lenses form a low-permeability layer, separating the overlying homogeneous fine to medium beach sands from the underlying poorly sorted coarse to medium sandy glacial deposits.^19^ For the upper layer, hydraulic conductivity was estimated based on grain size distribution measurements, using the empirical relationship by Hazen,^32^ indicating a consistent decrease in conductivity with depth in the upper meter (Figure S1). Values from June 2022 were used as representative conditions, capturing this stratification, and the model assumes a depth-dependent function (eq S4) fitted to this data (Figure 2c, *R* ^2^ = 0.89). Ranges of *K*_h_ of the tidal flat sediments (layer 2) and glacial fluvial sediments (layer 3) were estimated with slug tests conducted in the field in a multilevel well near the high-water line in 6 m, 12 m, 18 m, and 24 m depths.^33^ These estimations were complemented by centimeter-scale Darcy experiments and sieve analysis of the sediment core material collected during well installation. Based on this field data, the hydraulic conductivity in the model was adjusted to 6.25 m d^–1^ for layer 2 and 46 m d^–1^ for layer 3, respectively.

Water that enters the beach aquifer from the beach–face boundary carries air-saturated O_2_ concentrations of 225 μmol l^–1^ in the summer and 310 μmol l^–1^ in the winter simulation (temperatures in summer and winter were 22.2 and 6.7 °C, respectively, and the salinity was 32 PSU). Additionally, we implemented aeration with atmospheric O_2_ to account for gas exchange in the periodically desaturated upper layer, assuming rapid equilibration with atmospheric levels (see also eq S5). In the model, aeration was activated as a source term when saturation dropped below a threshold of 0.97, triggering a rapid increase in porewater O_2_ levels, while O_2_ consumption was allowed to occur simultaneously. To investigate the effect of aeration on O_2_ distribution, we additionally conducted simulations without this mechanism (Figure 4c,d). The O_2_ consumption rates in the model were derived from direct incubation measurements that are presented in our previous study.^16^ A depth-dependent function (see also eq S6) was fitted to measure O_2_ consumption rates in the upper 70 cm (Figure 2d, *R* ^2^ = 0.76 in winter and 0.99 in summer) that was used to extrapolate the rates to greater depths. Simulations were performed until a quasi-steady-state O_2_ distribution was reached during a tidal cycle, indicated by a difference of less than 0.1% in oxic volume between 2 subsequent tidal cycles.

## 3 Results

### 3.1 Oxygen Distribution in the Shallow Beach

We first examined the model results for the shallow intertidal beach to emphasize the effect of the topography on the flow pattern and to compare the model outcome with the measured O_2_ distribution. Oxic seawater infiltrated at the beach face of the intertidal beach aquifer. In summer, 47% of the intertidal beach area experienced net infiltration conditions (positive average boundary flux) during a tidal cycle (41% in winter), whereas net exfiltration was found for the remaining area. The infiltration zone is situated in the upper intertidal zone, starting at the HWL (Figure 3a,b). In the summer scenario, this zone was divided by an additional exfiltration zone due to a slight topographic depression ∼50–65 m from the mHWL, resulting in two distinct infiltration zones. In general, a reduction or a reversal of the beach slope was associated with a reduction in the level of the underlying O_2_ concentration, which we attribute to a reduced hydraulic gradient and thus a slowdown of porewater flow. Near the HWL, porewater flow paths were predominantly vertical, but the horizontal component increased with the distance to the HWL, aligning more with the horizontal beach topography. Consequently, horizontal cross-shore O_2_ gradients formed along the beach profile. In the summer scenario, horizontal oxic–anoxic transition zones emerged in the upper layer of the infiltration zone between ∼40 and 50 m and ∼80 and 90 m from the mHWL (Figure 3a). In winter, these gradients were found between ∼25 and 35 m and ∼65 and 75 m (Figure 3b).

**Figure 3:**
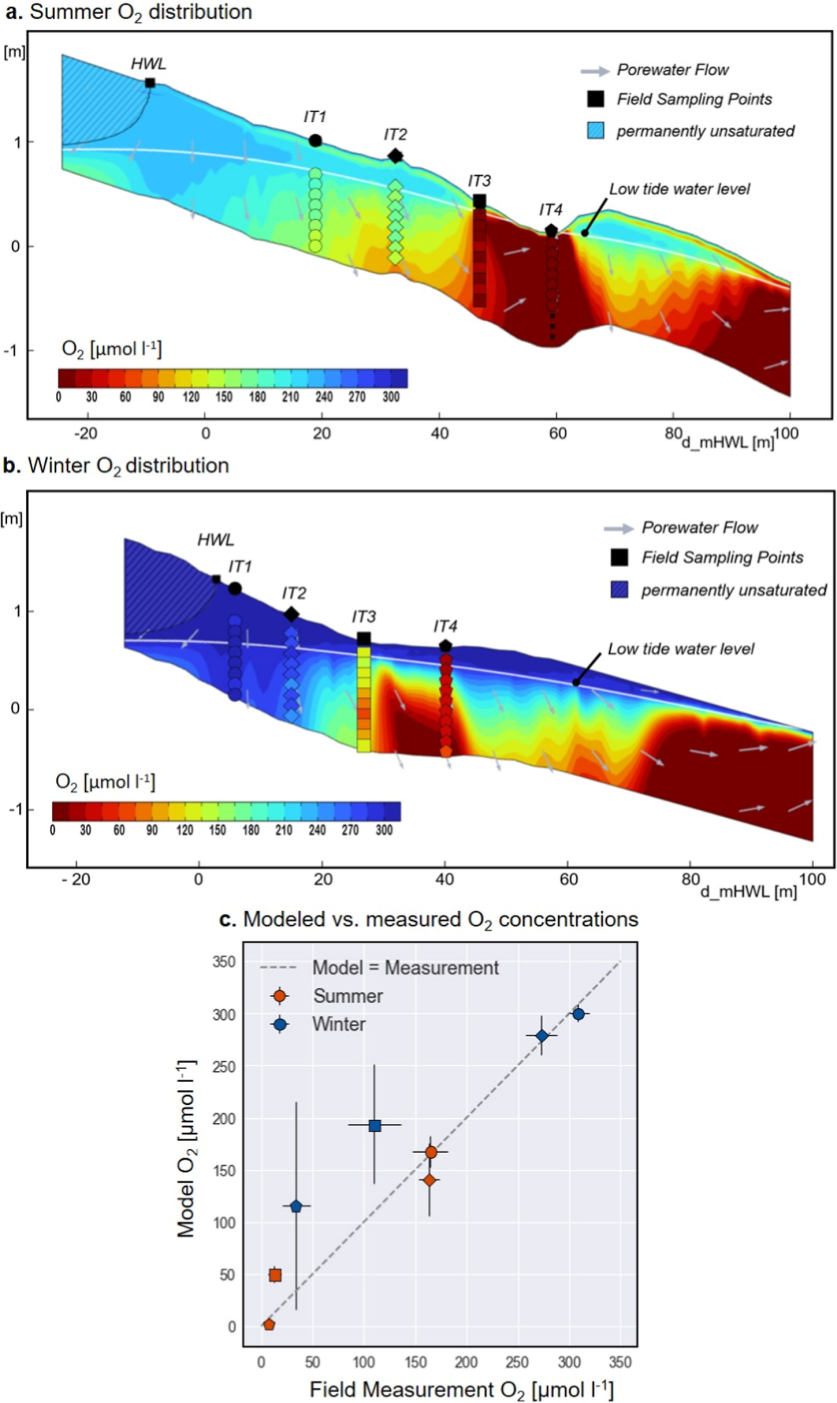
(a,b) Modeled tide-averaged quasi-stationary O_2_ distribution for the summer and winter case in the shallow intertidal beach aquifer infiltration zone, shown as color code. Arrows indicate pore water flow direction. The hatched areas indicate permanently unsaturated sediments. The white line indicates the water level during low tide (minimum water level), with the periodically desaturated zone above. Sampling locations at stations from previous sampling campaigns show measured concentrations in color code. Note: the station labels (IT1–4) refer to positions based on field sampling points, which varied slightly between campaigns and do not represent fixed wells. (c) Comparison of station mean values of modeled and measured O_2_ concentrations. The sampling station symbols correspond to the contour plots above. The error bars indicate the standard deviation.

The modeled horizontal O_2_ gradients were consistent with field data from four sampling stations (IT 1–4) along the upper transect of the infiltration zone (Figure 3a,b). A comparison of modeled and measured O_2_ concentrations showed a strong correlation, with the model reproducing the measured concentrations (Figure 3c, *R* ^2^ of 0.91 in summer and 0.73 in winter for fit of the “model = measurement” line to data). The model suggested vertical O_2_ gradients at some sampling stations (IT 2 in summer; IT 3 and IT 4 in winter) that were not observed in the field, and it overestimated O_2_ levels at IT 3 and IT 4 in winter (Figure 3a,b). Nevertheless, despite the complex flow patterns and topography, the measured values were well represented by the model.

### 3.2 Oxygen Distribution in the Deep USP

In summer, the seawater infiltration rate was 7.73 m^3^ per m shoreline and day and decreased by 35% to 5.08 m^3^ m^–1^ d^–1^ in winter. The resulting volume of the oxic zone (in this study, defined as O_2_ > 1 μmol L^–1^) extended over 711 m^3^ m^–1^ in the summer, averaged over a tidal cycle and excluding the permanently unsaturated sediments above the HWL (Figure 4a). In winter, the oxic zone increased to 849.6 m^3^ m^–1^ (Figure 4b). Thus, a volume of 138.6 m^3^ m^–1^ experienced seasonally varying redox conditions. Vertically, the oxic zone reached its maximum extent below the HWL, where maximum vertical porewater velocities are reached. O_2_ reached a maximum of 11.7 m in summer, increasing to 16.6 m in winter (Figure 4e,f). O_2_ concentrations started to drop below 5 μmol l^–1^ at 11 m depth in summer and 15.7 m depth in winter, marking the oxic–anoxic transition zone. Field data from a multilevel sampling well (ML2 at 6, 12, and 18 m depth) near the mHWL at Spiekeroog beach confirmed the seasonal position of this transition (Figure 4e,f).

**Figure 4:**
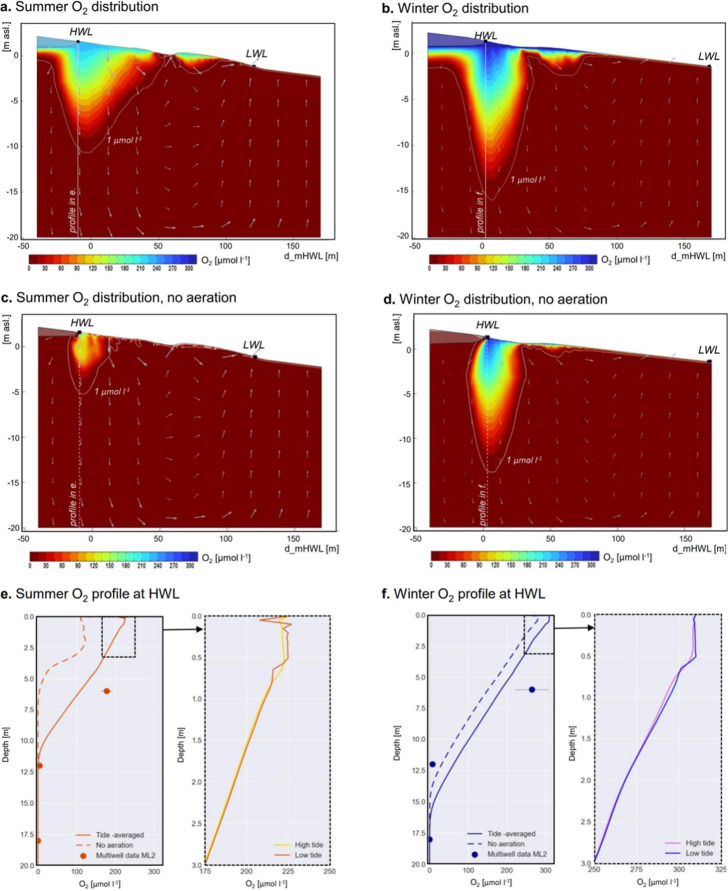
(a,b) Modeled tide-averaged quasi-stationary O_2_ distribution for the summer and winter case in the upper 20 m of the beach aquifer from −40 to 170 m distance to mHWL, shown as color code. Arrows indicate pore water flow direction. The shaded areas indicate permanently unsaturated sediments. White line depicts contour of 1 μmol L^–1^ indicating the oxycline. (c,d) Same as (a,b) but without the aeration function in desaturated sediment. (e,f) Modeled tide averaged O_2_ profiles at the HWL for the respective model case. Dashed area shows a zoom of the upper 3 m and compares the O_2_ profile at high and low tides for the model case including aeration. The dots show mean seasonal O_2_ concentrations measured in the multilevel well (ML2) near the mHWL at 6, 12, and 18 m depths with *n* = 3 values for summer 2022 (June–September) and winter 2022/23 (December–February). The error bars indicate the standard deviation.

In addition to seasonal variations, O_2_ concentrations fluctuated throughout a tidal cycle. Depth profiles of the first 3 m at the HWL showed tidal variations of up to 9.6 μmol l^–1^ in summer (Figure 4e) and 7.3 μmol l^–1^ in winter (Figure 4f). The greatest variations were observed just below the variably saturated layer at approximately 70 cm depth. Tidal flow dynamics also caused a corresponding fluctuation of the oxic volume by 9.4 m^3^ per m shoreline in summer and 8 m^3^ m^–1^ in winter. However, the tidal fluctuations were minor compared with seasonal variations.

### 3.3 Periodic Sediment Desaturation and Aeration with Atmospheric O_2_

At low tide, the desaturated layer had a maximum thickness of 65–68 cm at the HWL, gradually decreasing downslope toward the exfiltration zone (Figure 3a,b). The desaturated volume in the intertidal range amounts to 18.8 m^3^ m^–1^ in winter and 27 m^3^ m^–1^ in summer. As presented in the model description, we included the aqueous and aqueous O_2_ from the atmosphere in this layer. The impact of this process was tested by simulations excluded this aeration. The effect was significant especially in summer, where the oxic volume was reduced by 75% to 178.9 m^3^ m^–1^, and the O_2_ penetration depth decreased by 43% to 6.7 m. In winter, the effect was less pronounced with a O_2_ volume reduction by 37% to 531.7 m^3^ m^–1^ and a decrease in O_2_ penetration depth by 10% to 15 m (Figure 4c–f).

### 3.4 Oxygen Supply and Carbon Flux

Seawater infiltration rates and the resulting O_2_ supply from air-saturated seawater were higher in summer than in winter (Table 1). As tidal and wave forcing remained constant, this difference can be attributed to the more reflective and slightly steeper summer beach profile (Figure 2), leading to a 10% increase in O_2_ inflow despite the lower O_2_ concentration in summer seawater. In addition to the supply of O_2_ by infiltration, the aeration of the periodically desaturated layer provided significant amounts of O_2_, contributing 63 and 29% of the total O_2_ supply in summer and winter, respectively. Overall, the total O_2_ supply was more than double in summer compared to winter. The model showed no outflow of water containing dissolved O_2_, indicating that all supplied O_2_ is fully consumed. Hence, the O_2_ supply reflects the O_2_ net flux. O_2_ fluxes were used to estimate total aerobic organic carbon (OC) remineralization in the USP, assuming a respiration quotient C/O_2_ of 106:138, 106 mol O_2_ for OC oxidation, 32 mol O_2_ for nitrification, based on OM with a C/N ratio of 106:16.^34^ The aerobic OC remineralization in the USP amounts to 43 and 21 g of C per m shoreline and day in summer and winter, respectively.

**Table 1:** Model Results for Seasonal Seawater Infiltration Rates, Oxic Volume, O_2_ Supply, and Resulting Aerobic OC Remineralization for the USP

model output	value summer	value winter
infiltration rate [m^3^ m^–1^ d^–1^]	7.73	5.08
oxic volume [m^3^ m^–1^]	711.0	849.6
O_2_ supply infiltration [mol m^–1^ d^–1^]	1.74	1.58
O_2_ supply aeration [mol m^–1^ d^–1^]	2.90	0.66
total O_2_ supply/flux [mol m^–1^ d^–1^]	4.64	2.24
aerobic OC mineralization [g C m^–1^ d^–1^]	42.9	20.7

## 4 Discussion

### 4.1 Model Setup

Reactive transport models of beach aquifers commonly employ reaction and OC remineralization rates based on both literature values and model-derived rates, as established by Spiteri et al.^35^ These rates have been subsequently adopted and modified in various studies.^13,36–39^ To the best of our knowledge, approaches based on directly measured reaction rates have not been explicitly investigated in beach aquifer models. Here, we present a modeling approach that integrates measured O_2_ consumption rates as input variable and measured O_2_ concentrations as control, using O_2_ as a reactive tracer for OC remineralization. In this way, it was possible to consider the strong vertical gradient of reaction rates and pronounced seasonality. This represents a refinement compared to beach aquifer models that assume a constant OC remineralization rate over space and time. With regard to porewater transport, detailed geological investigations allowed for improved estimates of the hydraulic conductivity. Overall, the parametrization allowed our model to effectively replicate field-measured O_2_ concentrations (Figures 3 and 4e,f), providing a robust basis for evaluating O_2_ dynamics, total O_2_ uptake, and the resulting OC remineralization in the beach bioreactor. Notably, our model highlights, for the first time, the role of the periodic desaturation zone as a dynamic boundary layer for biogeochemical processes.

The presented model is a simplified representation of a complex natural system, simulating tide- and wave-driven groundwater flow and reactive O_2_ transport in an intertidal beach aquifer. Density-driven flow is not included to reduce computational demands, yet the model effectively reproduces the observed O_2_ concentrations. We implemented a fixed tidal amplitude and averaged wave forcing, a homogeneous distribution of hydraulic conductivity within each of the three model layers, and a stationary topography. In reality, factors such as spring–neap tidal cycles,^13,40^ varying wave action,^36,39^ storm floods,^14,20^ geological heterogeneity,^41,42^ morphodynamics,^14,20^ and varying freshwater influx^13,43^ influence solute distribution in beach aquifers. In particular, storm-induced morphological changes can shift the position of the USP and alter flow paths.^20^ Nevertheless, the reactive surface layer rapidly reestablishes due to fast POC turnover and constant seawater inflow, making it relatively insensitive to such disturbances.^16^ Deeper O_2_ distributions adjust more slowly, which may cause transient redox shifts beyond the seasonal patterns resolved here.^14^ These factors, which are not accounted for here, represent a limitation that may contribute to discrepancies between modeled and measured concentrations.

The model aims to integrate available, relevant data to represent the overall reactive transport of O_2_ for the Spiekeroog beach aquifer while balancing model simplicity with computational efforts. Consequently, the parametrization does not seek to capture every detail of reality. We acknowledge that other parameter combinations, particularly for hydraulic conductivity, may yield closer approximations, especially when incorporating additional tracers beyond O_2_. Improving our model approach would require more O_2_ rate and concentration measurements. Based on the current parametrization, O_2_ consumption rates at depth can be as low as 0.1 μmol L^–1^ h^–1^ in winter and 0.19 μmol L^–1^ h^–1^ in summer. Therefore, future experimental setups should be designed to capture these low rates accurately.

### 4.2 Controls of Oxygen Distribution

The simulated O_2_ distribution in the beach aquifer is controlled by the balance between the O_2_ supply, driven by seasonal seawater infiltration and the O_2_ solubilities as well as aeration with atmospheric O_2_ and O_2_ consumption, which is controlled by the availability of labile organic matter. The modeled seawater infiltration rates are consistent with previously reported values for Spiekeroog beach (4.5–12 m^3^ m^–1^ d^–1^).^44^ Seasonal changes in beach topography influence the infiltration, with steeper, reflecting profiles, typically associated with summer months,^28^ enhancing seawater infiltration, and increasing O_2_ supply compared to winter (Table 1). Our model suggests that this enhanced infiltration compensates lower seawater O_2_ concentrations in summer, resulting in greater overall O_2_ supply through seawater infiltration and mitigating seasonal redox shifts in the USP. This phenomenon, previously indicated by generic reactive transport modeling,^14^ implies that in the absence of seasonal topography changes, seasonal biogeochemical boundary conditions would induce more pronounced redox shifts at depth. Compared to the impact of seasonal changes in infiltration rates, O_2_ fluctuations associated with tidal volume flows are relatively minor, as supported by field observations (Figure S2), and are less pronounced than those reported by Charbonnier et al.^45^ at the more energetic French Aquitaine Coast.

Despite enhanced infiltration in summer, we observed a considerable seasonal upward shift of the oxycline, indicating that O_2_ consumption has a strong impact on O_2_ distribution. In winter, the modeled oxycline deepens by 50% relative to summer. Multilevel well data near the HWL confirm this seasonal trend, showing fluctuating O_2_ concentrations at 6 m depth with minima in summer, and a redox transition from oxic to anoxic conditions between 9 and 15 m (Figure 4).^17^ Similar seasonal O_2_ fluctuations have been reported for other shallow beach aquifers, including the French Aquitaine Coast^11^ and the Delaware Beach Aquifer.^46^ Our model connects the upper reactive layer with the deeper USP, showing how seasonal biogeochemical conditions in the upper infiltration zone, such as the seasonal O_2_ consumption, can propagate to greater depths, causing seasonal oxycline shifts by several meters.

However, the effects of seasonal O_2_ consumption would be many times greater if a large part of the O_2_ demand in the upper layer could not be met by the supply of atmospheric O_2_ provided by periodic sediment desaturation and aeration during low tide. The role of the periodically desaturated layer was previously discussed by Schutte et al.,^23^ who reported atmospheric O_2_ supporting nitrification, and Geng et al.,^24^ who demonstrated significant biodegradation of organic contaminants in this layer. Here, we highlight that the periodically desaturated layer coincides with the retention layer for fresh marine POC, and it is thus characterized by both high O_2_ demand and efficient O_2_ supply. The dynamic aeration mitigates the impact of the highly reactive top layer on the supply of the O_2_ to deeper layers, dampening the imprint of the seasonal OM input on the deeper beach reactor. Assuming that O_2_ introduced by aeration is primarily used for the respiration of retained POC, O_2_ from infiltrating seawater is rapidly transported together with DOC through the upper layer and remains available in the deeper reactor. This process maintains the O_2_ supply to the deeper subsurface while facilitating the rapid degradation of reactive OC, accounting for up to 30–60% of total O_2_ consumption (Table 1). Without the aeration of the upper layer, the oxic zone would be reduced by 75% in summer and 37% in winter (Figure 4). Ultimately, this aeration mechanism explains the apparent anomaly of the beach reactor: intense carbon remineralization in the top layer but a deep oxycline below.

### 4.3 Organic Carbon Remineralization and Mass Balance

Thus far, published estimates of the organic carbon turnover in beach aquifers are typically based on the porewater analysis of dissolved inorganic carbon.^38,47,48^ We translated our modeled O_2_ consumption, which is based on direct rate measurements, into aerobic OC remineralization (see also Section 3.4), assuming that OC remineralization is the dominant O_2_ sink in the oxic zone. The estimated OC remineralization leads to a production of 3.6 and 1.7 mol DIC m^–1^ d^–1^ in summer and winter, respectively, and compares well to estimated DIC flux from the beach aquifer to the ocean of ∼4 mol m^–1^ d^–1^ reported by Charbonnier et al. (2022) for the French Aquitaine Coast. Most aerobic OC remineralization occurs in the upper layer. In the field study that served as the basis for this model study, we estimated OC remineralization in the upper layer of the infiltration zone to amount to 34 and 9 g C m^–1^ d^–1^ for the selected campaigns in summer (June campaign) and winter (December campaign), respectively.^16^ The model confirms these values (34 and 12 g C m^–1^ d^–1^) and further indicates that this layer accounts for ∼80% (summer) and ∼57% (winter) of total aerobic remineralization (43 and 21 g C m^–1^ d^–1^; see Table 1), highlighting its key role in OM turnover in beach aquifers.

Overall, the total OC turnover is intense, especially in summer, where the rate per area (0.7 g C m^–2^ d^–1^) is more than twice the rates observed in adjacent subtidal sands (up to 0.3 g C m^–2^ d^–1^)^49^ and 10-fold times higher than those in nearby muds (up to 0.07 g C m^–2^ d^–1^).^50^ What are the OC sources that fuel such high rates? In a first step, we assume that OC remineralization in the USP is fueled by both dissolved and particulate OM introduced by infiltrating seawater.^11,37^ At the site, seawater DOC concentrations range from 180 μmol L^–1^ in summer to 140 μmol L^–1^ in winter (unpublished data from seawater analysis during the summer (June) and winter (December) 2022 campaign; samples are measured using high-temperature catalytic oxidation on a Shimadzu TOC-VCPH), while the DOC concentration in the USP outflow is ∼80 μmol l^–1^,^17^ representing unreactive DOC. Applying the seawater infiltration rates (Table 1), we can calculate that the USP is supplied by 9.3 and 3.7 g of reactive DOC m^–1^ d^–1^ in summer and winter, respectively. The difference in the total OC remineralization suggests that the remaining contribution must come from particulate OM, requiring an input of 33.6 and 17 g of reactive POC m^–1^ d^–1^. Based on the infiltration rates, this implies theoretical POC concentrations of 4.3 g m^–3^ in summer and 3.4 g m^–3^ in winter. These values are notably higher than typical POC levels reported for the open North Sea, which range between 0.01 and 0.5 g m^–3^, and thus introduce uncertainty into our mass balance calculations.^51–53^

The gap in the OC supply could be explained either by a significant contribution of the total standing stock of sedimentary OC (TOC), which may be less refractory than assumed, or by mechanisms of POC enrichment due to wave motion as the seawater is transported from the foreshore to the swash zone. Assuming TOC as the missing OC source, we roughly calculate that a TOC content of 0.01 wt %^16^ could sustain the OC turnover for at least 8 years. However, no trend in TOC with depth has been observed, despite the fact that sediments at a few meters depth are of the order of hundred years old.^54^ Assuming POC enrichment, we speculate that in shallow sandy coastal regions, fine particles remain suspended in the water column instead of settling on the seafloor, resulting in a net shoreward transport by waves and currents,^55^ and an enrichment of POC in infiltrating waters compared to open-water conditions where POC is typically measured. Indeed, in our previous field study, we found a labile POC fraction of 1.6 to 5.8 mg L_Sed_^–1^ in the 15 cm thick infiltration layer that is rapidly consumed with an estimated turnover time of ∼3 days.^16^ With the given infiltration rates, we calculate that the POC concentrations of the infiltrated seawater are in the range of 0.8 to 2.9 g m^–3^, indicating POC enrichment above average seawater concentrations. Temporarily increasing POC inputs due to algal blooms^56^ and beach wrack deposition^57^ can further contribute substantial OC inputs to the system.^57^ However, in the previous field study,^16^ we found no indication of beach wrack deposition in the sediment samples. Although these combined processes may plausibly account for the elevated POC inputs during seawater infiltration, the current literature on such POC supply mechanisms to high-energy beaches remains limited. We therefore identify the elevated POC concentrations required to meet the modeled OC fluxes as a priority for targeted future studies to clarify supply and enrichment mechanisms and quantify these inputs.

### 4.4 Environmental Implications

In our previous study,^16^ we revealed a strong correlation between O_2_ consumption and the seasonal OC availability in the infiltration zone, indicating that the beach reactor is primarily limited by labile OC. Building on these findings, the imposed seasonal O_2_ consumption in our model demonstrates how the oxycline responds to OM inputs, with cascading effects on the deeper subsurface. About 139 m^3^ per m shoreline of the beach aquifer is subject to variable redox conditions, determining the metabolism of the microbial community. We assume that heterotrophic metabolisms dominate the upper USP, especially in summer when OM inputs are elevated. During this season, a shallow oxycline and expanded anoxic layers create potential for reoxidation when the oxycline shifts downward in winter, also increasing potential for chemolithotrophic pathways.

Redox shifts in coastal sands have broad environmental implications. The frequent alternation between oxic and anoxic conditions changes redox states of both dissolved and solid-phase compounds, such as iron and manganese, facilitating phosphorus release^58^ and impacting adsorption/desorption dynamics of DOM on minerals.^59,60^ In these systems, the oxic–anoxic transition zone is a hotspot for nitrogen cycling^61^ and also influences redox-sensitive contaminants such as arsenic, which are either released or scavenged depending on prevailing redox conditions.^62^ Additionally, shifting oxic–anoxic interfaces generate reactive O_2_ species,^63^ with the potential to break refractory OC into more labile forms, enhancing the overall reactivity of the beach reactor. It should be noted that the intense OM turnover and the deep oxygen penetration suggest increased nitrate production via nitrification. Modeled O_2_ consumption supports nitrate production of 0.5–1 mol m^–1^ d^–1^ (winter–summer), which serves as an electron acceptor for denitrification at greater depths. Thus, the distribution of dissolved reactants, nutrients, and contaminants within the beach bioreactor and the corresponding exchange fluxes with coastal waters are not constant but strongly depend on the seasonal supply of OM to the carbon-limited bioreactor.

Our study emphasizes that variable boundary conditions and OM inputs as well as the periodically desaturated layer within the infiltration zone drive significant changes in redox conditions and determine the aerobic OC turnover in beach aquifers. Yet, the sources and transport mechanisms of OC to the shallow and deep beach aquifer remain unclear. Importantly, further investigations of biogeochemical reaction rates in the deeper subsurface as well as targeted investigations into the OC sources fueling these processes are needed to fully elucidate the functioning of the beach aquifer as a biogeochemical reactor.

## Supplementary Material



## Data Availability

Model output data are publicly available on ZENODO via 10.5281/zenodo.14356912.

## Notes

Details on the numerical model setup and boundary conditions for the transport and reactive stage of the model; transport model parameters, reactive model parameters, hydraulic conductivity estimates for field campaigns May–August 2022, and comparison of continuous in situ O_2_ measurements and model during several tidal cycles in December 2022 (PDF)

## References

[1] Moore W. S. (1999). The subterranean
estuary: a reaction zone of ground water and sea water. Mar. Chem..

[2] Anschutz P., Smith T., Mouret A., Deborde J., Bujan S., Poirier D., Lecroart P. (2009). Tidal sands
as biogeochemical reactors. Estuarine, Coastal
and Shelf Science.

[3] Wilson S. J., Moody A., McKenzie T., Cardenas M. B., Luijendijk E., Sawyer A. H., Wilson A., Michael H. A., Xu B., Knee K. L., Cho H., Weinstein Y., Paytan A., Moosdorf N., Chen C. A., Beck M., Lopez C., Murgulet D., Kim G., Charette M. A., Waska H., Ibánhez J. S.
P., Chaillou G., Oehler T., Onodera S., Saito M., Rodellas V., Dimova N., Montiel D., Dulai H., Richardson C., Du J., Petermann E., Chen X., Davis K. L., Lamontagne S., Sugimoto R., Wang G., Li H., Torres A. I., Demir C., Bristol E., Connolly C. T., McClelland J. W., Silva B. J., Tait D., Kumar B., Viswanadham R., Sarma V., Silva-Filho E., Shiller A., Lecher A., Tamborski J., Bokuniewicz H., Rocha C., Reckhardt A., Böttcher M. E., Jiang S., Stieglitz T., Gbewezoun H. G. V., Charbonnier C., Anschutz P., Hernández-Terrones L. M., Babu S., Szymczycha B., Sadat-Noori M., Niencheski F., Null K., Tobias C., Song B., Anderson I. C., Santos I. R. (2024). Global subterranean estuaries modify
groundwater nutrient loading to the ocean. Limnol
Oceanogr Letters.

[4] Santos I. R., Burnett W. C., Dittmar T., Suryaputra I. G. N. A., Chanton J. (2009). Tidal pumping drives nutrient and
dissolved organic
matter dynamics in a Gulf of Mexico subterranean estuary. Geochim. Cosmochim. Acta.

[5] Santos I. R., Chen X., Lecher A. L., Sawyer A. H., Moosdorf N., Rodellas V., Tamborski J., Cho H.-M., Dimova N., Sugimoto R., Bonaglia S., Li H., Hajati M.-C., Li L. (2021). Submarine groundwater discharge impacts
on coastal nutrient biogeochemistry. Nat. Rev.
Earth Environ.

[6] Santos I. R., Eyre B. D., Huettel M. (2012). The driving
forces of porewater and
groundwater flow in permeable coastal sediments: A review. Estuarine, Coastal and Shelf Science.

[7] Huettel M., Berg P., Kostka J. E. (2014). Benthic Exchange
and Biogeochemical
Cycling in Permeable Sediments. Annu. Rev. Mar.
Sci..

[8] Probandt D., Eickhorst T., Ellrott A., Amann R., Knittel K. (2018). Microbial
life on a sand grain: from bulk sediment to single grains. ISME J..

[9] Ahmerkamp S., Marchant H. K., Peng C., Probandt D., Littmann S., Kuypers M. M. M., Holtappels M. (2020). The effect
of sediment grain properties
and porewater flow on microbial abundance and respiration in permeable
sediments. Sci. Rep..

[10] Robinson C., Gibbes B., Li L. (2006). Driving mechanisms for groundwater
flow and salt transport in a subterranean estuary. Geophys. Res. Lett..

[11] Charbonnier C., Anschutz P., Poirier D., Bujan S., Lecroart P. (2013). Aerobic respiration
in a high-energy sandy beach. Mar. Chem..

[12] Seidel M., Beck M., Greskowiak J., Riedel T., Waska H., Suryaputra Ig. N. A., Schnetger B., Niggemann J., Simon M., Dittmar T. (2015). Benthic-pelagic
coupling of nutrients
and dissolved organic matter composition in an intertidal sandy beach. Mar. Chem..

[13] Heiss J. W., Post V. E. A., Laattoe T., Russoniello C. J., Michael H. A. (2017). Physical Controls on Biogeochemical
Processes in Intertidal
Zones of Beach Aquifers. Water Resour. Res..

[14] Greskowiak J., Seibert S. L., Post V. E. A., Massmann G. (2023). Redox-zoning
in high-energy
subterranean estuaries as a function of storm floods, temperatures,
seasonal groundwater recharge and morphodynamics. Estuarine, Coastal and Shelf Science.

[15] Huettel M., Rusch A. (2000). Transport and degradation
of phytoplankton in permeable sediment. Limnol.
Oceanogr..

[16] Auer F., Ahmerkamp S., Cueto J., Winter C., Holtappels M. (2025). Extensive
Oxygen Consumption in the Intertidal Infiltration Zone of Beach Aquifers—The
Impact of Seasonal Input, Filtration Efficiency, and Morphodynamics. Journal of Geophysical Research: Biogesciences.

[17] Reckhardt A., Meyer R., Seibert S. L., Greskowiak J., Roberts M., Brick S., Abarike G., Amoako K., Waska H., Schwalfenberg K., Schmiedinger I., Wurl O., Böttcher M. E., Massmann G., Pahnke K. (2024). Spatial and
temporal dynamics of groundwater biogeochemistry in the deep subsurface
of a high-energy beach. Mar. Chem..

[18] Massmann G., Greskowiak J., Degenhardt J., Engelen B., Holtappels M., Meyer R., Müller-Petke M., Moosdorf N., Niggemann J., Pahnke K., Post V., Reckhardt A., Schwalfenberg K., Seibert S., Waska H., Winter C. (2025). High Energy
Systems are Underrepresented in Global Porewater Studies of Sandy
Beach Aquifers. Estuarine, Coastal and Shelf
Science.

[19] Massmann G., Abarike G., Amoako K., Auer F., Badewien T. H., Berkenbrink C., Böttcher M. E., Brick S., Cordova I. V. M., Cueto J., Dittmar T., Engelen B., Freund H., Greskowiak J., Günther T., Herbst G., Holtappels M., Marchant H. K., Meyer R., Müller-Petke M., Niggemann J., Pahnke K., Pommerin D., Post V., Reckhardt A., Roberts M., Schwalfenberg K., Seibert S. L., Siebert C., Skibbe N., Waska H., Winter C., Zielinski O. (2023). The DynaDeep observatory—a
unique approach to study high-energy subterranean estuaries. Front. Mar. Sci..

[20] Greskowiak J., Massmann G. (2021). The impact of morphodynamics
and storm floods on pore
water flow and transport in the subterranean estuary. Hydrological Processes.

[21] Ahrens J., Beck M., Marchant H. K., Ahmerkamp S., Schnetger B., Brumsack H. (2020). Seasonality of Organic
Matter Degradation
Regulates Nutrient and Metal Net Fluxes in a High Energy Sandy Beach. J. Geophys. Res. Biogeosci..

[22] Cogswell C., Heiss J. W. (2021). Climate and Seasonal Temperature Controls on Biogeochemical
Transformations in Unconfined Coastal Aquifers. JGR Biogeosciences.

[23] Schutte C. A., Wilson A. M., Evans T., Moore W. S., Joye S. B. (2018). Deep oxygen
penetration drives nitrification in intertidal beach sands. Limnol. Oceanogr..

[24] Geng X., Boufadel M. C., Cui F. (2017). Numerical
modeling of subsurface
release and fate of benzene and toluene in coastal aquifers subjected
to tides. Journal of Hydrology.

[25] Geng X., Heiss J. W., Michael H. A., Li H., Raubenheimer B., Boufadel M. C. (2021). Geochemical fluxes in sandy beach
aquifers: Modulation
due to major physical stressors, geologic heterogeneity, and nearshore
morphology. Earth-Sci. Rev..

[26] COMSOL , COMSOL Multiphysics V. 6.1, 2022, www.comsol.com (accessed Aug 28, 2024).

[27] Bundesamt für Seeschifffahrt und Hydrographie (BSH) [Federal Maritime and Hydro-graphic Agency] , Wellenradar Nordergründe, 2023, https://www.bsh.de/DE/DATEN/Klima-und-Meer/Meeresumweltmessnetz/_Module/Info_Stationen/info_Nordergruende_Wellenradar_node.html (accessed Nov 13, 2023).

[28] Wright L.
D., Short A. D. (1984). Morphodynamic
variability of surf zones and beaches:
A synthesis. Marine Geology.

[29] Grünenbaum N., Greskowiak J., Sültenfuß J., Massmann G. (2020). Groundwater
flow and residence times below a meso-tidal high-energy beach: A model-based
analyses of salinity patterns and 3H-3He groundwater ages. Journal of Hydrology.

[30] Beck M., Reckhardt A., Amelsberg J., Bartholomä A., Brumsack H.-J., Cypionka H., Dittmar T., Engelen B., Greskowiak J., Hillebrand H., Holtappels M., Neuholz R., Köster J., Kuypers M. M. M., Massmann G., Meier D., Niggemann J., Paffrath R., Pahnke K., Rovo S., Striebel M., Vandieken V., Wehrmann A., Zielinski O. (2017). The drivers
of biogeochemistry in
beach ecosystems: A cross-shore transect from the dunes to the low-water
line. Mar. Chem..

[31] Meyer R., Greskowiak J., Seibert S. L., Post V. E., Massmann G. (2025). Effects of
boundary conditions and aquifer parameters on salinity distribution
and mixing-controlled reactions in high-energy beach aquifers. Hydrol. Earth Syst. Sci..

[32] Hölting, B. ; Coldewey, W. G. Hydrogeology; Springer Berlin Heidelberg, 2019. 10.1007/978-3-662-56375-5.

[33] Meyer R., Reckhardt A., Greskowiak J., Seibert S., Skibbe N., Sültenfuß J., Massmann G. (2025). Water bodies mix dynamically
and residence times are between days and decades in the subterranean
estuary of a high energy beach. ESS Open Archive.

[34] Redfield, A. C. ; Ketchum, B. H. ; Richards, F. A. The composition of seawater: Comparative and descriptive oceanography. In The sea: ideas and observations on progress in the study of the seas, 1963; pp 22–77.

[35] Spiteri C., Slomp C. P., Charette M. A., Tuncay K., Meile C. (2008). Flow and nutrient
dynamics in a subterranean estuary (Waquoit Bay, MA, USA): Field data
and reactive transport modeling. Geochim. Cosmochim.
Acta.

[36] Anwar N., Robinson C., Barry D. A. (2014). Influence
of tides and waves on the
fate of nutrients in a nearshore aquifer: Numerical simulations. Adv. Water Resour..

[37] Kim K. H., Heiss J. W., Michael H. A., Cai W., Laattoe T., Post V. E. A., Ullman W. J. (2017). Spatial Patterns
of Groundwater Biogeochemical
Reactivity in an Intertidal Beach Aquifer. J.
Geophys. Res. Biogeosci..

[38] Kim K. H., Heiss J. W., Michael H. A., Ullman W. J., Cai W.-J. (2022). Seasonal
and Spatial Production Patterns of Dissolved Inorganic Carbon and
Total Alkalinity in a Shallow Beach Aquifer. Front. Mar. Sci..

[39] Rakhimbekova S., Wu M. Z., Post V., Robinson C. E. (2022). Effect of time-varying
wave conditions on the fate of nitrogen in a freshwater unconfined
nearshore aquifer. Adv. Water Resour..

[40] Heiss J. W., Michael H. A. (2014). Saltwater-freshwater
mixing dynamics in a sandy beach
aquifer over tidal, spring-neap, and seasonal cycles. Water Resour. Res..

[41] Geng X., Michael H. A., Boufadel M. C., Molz F. J., Gerges F., Lee K. (2020). Heterogeneity Affects Intertidal Flow Topology in Coastal Beach Aquifers. Geophys. Res. Lett..

[42] Heiss J. W., Michael H. A., Koneshloo M. (2020). Denitrification hotspots in intertidal
mixing zones linked to geologic heterogeneity. Environ. Res. Lett..

[43] Goodridge B. M., Melack J. M. (2014). Temporal Evolution
and Variability of Dissolved Inorganic
Nitrogen in Beach Pore Water Revealed Using Radon Residence Times. Environ. Sci. Technol..

[44] Grünenbaum N., Ahrens J., Beck M., Gilfedder B. S., Greskowiak J., Kossack M., Massmann G. (2020). A Multi-Method Approach
for Quantification of In- and Exfiltration Rates from the Subterranean
Estuary of a High Energy Beach. Front. Earth
Sci..

[45] Charbonnier C., Anschutz P., Deflandre B., Bujan S., Lecroart P. (2016). Measuring
pore water oxygen of a high-energy beach using buried probes. Estuarine, Coastal and Shelf Science.

[46] Kim K. H., Michael H. A., Field E. K., Ullman W. J. (2019). Hydrologic
Shifts
Create Complex Transient Distributions of Particulate Organic Carbon
and Biogeochemical Responses in Beach Aquifers. J. Geophys. Res. Biogeosci..

[47] Liu Y., Jiao J. J., Liang W., Santos I. R., Kuang X., Robinson C. E. (2021). Inorganic
carbon and alkalinity biogeochemistry and
fluxes in an intertidal beach aquifer: Implications for ocean acidification. Journal of Hydrology.

[48] Charbonnier C., Anschutz P., Abril G., Mucci A., Deirmendjian L., Poirier D., Bujan S., Lecroart P. (2022). Carbon dynamics driven
by seawater recirculation and groundwater discharge along a forest-dune-beach
continuum of a high-energy meso-macro-tidal sandy coast. Geochim. Cosmochim. Acta.

[49] Ahmerkamp S., Winter C., Krämer K., Beer D. d., Janssen F., Friedrich J., Kuypers M. M. M., Holtappels M. (2017). Regulation
of benthic oxygen fluxes in permeable sediments of the coastal ocean. Limnol. Oceanogr..

[50] Müller D., Liu B., Geibert W., Holtappels M., Sander L., Miramontes E., Taubner H., Henkel S., Hinrichs K.-U., Bethke D., Dohrmann I., Kasten S. (2025). Depositional controls and budget
of organic carbon burial in fine-grained sediments of the North Sea—the
Helgoland Mud Area as a natural laboratory. Biogeosciences.

[51] Postma H., Rommets J. W. (1984). Variations of particulate
organic carbon in the central
North Sea. Netherlands Journal of Sea Research.

[52] Suratman S., Weston K., Jickells T., Fernand L. (2009). Spatial and seasonal
changes of dissolved and particulate organic C in the North Sea. Hydrobiologia.

[53] Chaichana S., Jickells T., Johnson M. (2019). Interannual
variability in the summer
dissolved organic matter inventory of the North Sea: implications
for the continental shelf pump. Biogeosciences.

[54] Seibert S. L., Böttcher M. E., Schubert F., Pollmann T., Giani L., Tsukamoto S., Frechen M., Freund H., Waska H., Simon H., Holt T., Greskowiak J., Massmann G. (2019). Iron sulfide formation in young and rapidly-deposited
permeable sands at the land-sea transition zone. Science of The Total Environment.

[55] Desmit X., Schartau M., Riethmüller R., Terseleer N., Van Der Zande D., Fettweis M. (2024). The transition between
coastal and
offshore areas in the North Sea unraveled by suspended particle composition. Science of The Total Environment.

[56] McAllister C. D., Parsons T. R., Stephens K., Strickland J. D. H. (1961). Measurements
of primary production in coastal sea water using a large-volume plastic
sphere. Limnology & Oceanography.

[57] Waska H., Banko-Kubis H. M. (2024). Dissolved organic matter released
from beach wrack
is source-specific and molecularly highly diverse. Biogeochemistry.

[58] Zhou Z., Henkel S., Kasten S., Holtappels M. (2023). The iron “redox
battery” in sandy sediments: Its impact on organic matter remineralization
and phosphorus cycling. Science of The Total
Environment.

[59] Linkhorst A., Dittmar T., Waska H. (2017). Molecular
Fractionation of Dissolved
Organic Matter in a Shallow Subterranean Estuary: The Role of the
Iron Curtain. Environ. Sci. Technol..

[60] Zhou Z., Waska H., Henkel S., Dittmar T., Kasten S., Holtappels M. (2024). Iron Promotes the Retention of Terrigenous
Dissolved
Organic Matter in Subtidal Permeable Sediments. Environ. Sci. Technol..

[61] Wu J., Hong Y., Wilson S. J., Song B. (2021). Microbial nitrogen
loss by coupled nitrification to denitrification and anammox in a
permeable subterranean estuary at Gloucester Point, Virginia. Mar. Pollut. Bull..

[62] Rakhimbekova S., O’Carroll D. M., Andersen M. S., Wu M. Z., Robinson C. E. (2018). Effect
of Transient Wave Forcing on the Behavior of Arsenic in a Nearshore
Aquifer. Environ. Sci. Technol..

[63] Van
Erk M. R., Bourceau O. M., Moncada C., Basu S., Hansel C. M., De Beer D. (2023). Reactive oxygen species affect the
potential for mineralization processes in permeable intertidal flats. Nat. Commun..

