# Supplementary material for: The Beach Bioreactor: Unraveling the Anomaly of Intensive Remineralization above a Deep Oxycline
Source: Environ Sci Technol. 2025 Sep 12;59(37):19835–45. doi: 10.1021/acs.est.5c05752 (PMC12461910; doi:10.1021/acs.est.5c05752)
Supplement: Supplementary file 1 [file es5c05752_si_001.pdf]

Supporting Information

The beach bioreactor: unraveling the anomaly of intensive remineralization above a deep oxycline

Felix Auer[1,], Janek Greskowiak[2], Rena Meyer[2], Anja Reckhardt[3] and Moritz Holtappels[1]*

[1]Alfred Wegener Institute Helmholtz Center for Polar and Marine Research, Am Handelshafen 12, 27570, Bremerhaven, Germany. [2] Institute of Biology and Environmental Sciences, Carl von Ossietzky Universität Oldenburg, Ammerländer Heerstraße 11, 26129 Oldenburg, Germany

[3]Institute for Chemistry and Biology of the Marine Environment (ICBM), Carl von Ossietzky Universität Oldenburg, Carl-von-Ossietzky-Straße 9-11, 26129 Oldenburg, Germany

* Corresponding author: felix.auer@awi.de

Pages: 8 (including this page)

Tables: 2

Figures: 2

1.1 Stage 1: Numerical Transport Model. At the beach-face boundary, the periodic pressure from tides and wave setup drives groundwater flow in the upper saline plume. This signal was included as a time (t)-dependent head boundary condition using equation S1, where A is the tidal amplitude, and η the wave setup as calculated from equation S2 after Nielsen [1] as follows

$$Head(t) = A \cdot cos(12.567 * t) \; + \; \eta(t) \tag{S1}$$

$$\eta(t) = \frac{0.4 \cdot H_{rms}}{1 + 10\,\frac{D + \,\eta(t)}{H_{rms}}} \tag{S2}$$

Here, D is the still water depth at location x and H_{rms} is the root mean square of the significant wave height H. This boundary condition was applied such that a pressure signal was induced only when the water level exceeded the topographic height of the beach at location x. If this condition was not met, a seepage face was present. No-flow conditions are set at the bottom and the sea-ward boundaries (Figure 2-a). The initial time step in the simulations was set to a 0.01 minutes, with a maximum step of 0.05 min during model runs, sufficient to resolve tidal fluctuations and transport dynamics. For convergence, we used COMSOL's built-in relative tolerance of 0.001 for the constant Newton solver and a damping factor of 0.95.

The resulting porewater flow from tidal and wave-induced pressure oscillations was simulated using Richards` equation [2] for variably saturated media, along with the van Genuchten retention model [3] to describe soil matrix parameters for medium sand [4] (**Error! Reference source not found.**). The porosity of 0.34 was based on salinity breakthrough curves in flow through

reactors (unpublished data) and is consistent with previous modelling at the site [5]. To accurately represent the position of the intertidal zone during both the summer and the winter scenario in the model, the high tide height was set according to the last high tide level preceding sampling [6]. High and low water lines in the presented model results correspond to maximum and minimum water levels during a tidal cycle (including wave setup), with the x-coordinate representing the distance from the mean high-water line (mHWL) at 1.37 m asl.

Hydraulic conductivity (Kh) was estimated from d10 of the measured grain size, using the empirical relationship by Hazen [7] at 15° C (equation S3). In the upper beach layer, the depth-dependent function fitted to the estimates of Kh included in the model is given in equation S4, where x is replaced by the horizontal hydraulic conductivity, K_h, in m d^{-1}. The function parameters a,b and c are given in **Error! Reference source not found.**. Vertical anisotropy factors for K_h were adopted from previous modeling studies at the site (Table S1) [5,8].

$$K_h\left[\frac{m}{s}\right] = 0.0133 \cdot (d_{10}\ [mm])^2 \tag{S3}$$

$$x = a \cdot e^{\left(\frac{b}{\left(Depth\left[\frac{1}{m}\right]+c\right)}\right)} \tag{S4}$$

Time-dependant simulations of groundwater flow were conducted over several tidal cycles, reaching a quasi steady-state flowfield by the sixth cycle. This flowfield was then used to represent groundwater flow during a tidal cycle in the reactive model.

Table S 1. Values for parameters used in the numerical transport model

**Transport Model Parameter**	**Value**
Q_{in}	0.51 m^3 m^{-1} d^{-1}
Density (ρ)	1000 kg m^{-3}
Tidal amplitude (A)	1.37 m
Mean significant wave height (H)	0.59 m
Flood tide height summer / winter case	1.43 / 1.16 m asl.
Soil matrix parameter α	18 m^{-1}
Soil matrix parameter n	1.4
Soil matrix parameter Θ_R	0
Porosity (Θ)	0.34
Hydraulic Conductivity layer 1 parameter a	19.4
Hydraulic Conductivity layer 1 parameter b	2.24
Hydraulic Conductivity layer 1 parameter c	1.75
Horizontal Hydraulic Conductivity (K_h) layer 2	6.25 m d^{-1}
Horizontal Hydraulic Conductivity (K_h) layer 3	46 m d^{-1}
Vertical Anisotropy all layers	2

1.2 Stage 2: Coupled Reactions. Transient simulations of reactive oxygen (O_2) transport were conducted by periodically coupling the porewater flowfield from Stage 1 with the advective transport and consumption of O_2. Air saturation of infiltrating seawater based on mean porewater temperature (22.2 ˚C in summer, 6.7 ˚C in winter [9]) and salinity of 32 PSU (Table 2), was set as a fixed concentration at the beach-face boundary. A concentration of 0 was set at the landward boundary and no-flux conditions at the bottom and the sea-ward boundaries (**Error! Reference source not found.**-a). The aeration rate of porewaters in the desaturated beach layer (when saturation fell below 0.97) is given in equation S5 and Table S2. This approach assumes a well-aerated unsaturated zone, where porewater O_2 concentrations rapidly equilibrate with atmospheric levels. Given that the atmosphere serves as a virtually infinite reservoir of O_2 and diffusion in air is significantly faster than in water, a fast, concentration-dependent rate was applied to increase porewater O_2 levels until equilibrium was reached.

$$R_{reair}(Sat.\ \leq 0.97\ ) = 0.1\ min^{-1} \cdot\ (O_{2,airsat} -\ O_2) \qquad \text{(S5)}$$

The depth-dependent function fitted to the field-measured O_2 consumption rates is given in equation S4, where x is replaced by the maximum O_2 consumption rate $R_{O2,max}$ in µmol l^{-1} h^{-1}. In winter, the function was fitted to the depth-averaged rates measured at the 4 stations at the infiltration zone transect ($R^2 = 0.76$). In summer, the function was fitted to the rate decrease measured at the uppermost station IT 1 ($R^2 = 0.99$), which exhibits a typical reactive top layer, while the other station (IT 2) in the upper infiltration zone shows an atypical temporary increase of rates at 20 cm depth. With depth, the function approaches the value of the parameter a, hence

this parameter represents O_2 consumption rates at depth. O_2 rates in the deeper subsurface are assumed to be very low, and thus far no data is available at the field site below 1 m. Therefore, parameter a was loosely calibrated against O_2 concentration data obtained from sampling wells at 6, 12, and 18 m depth near the mHWL [10] to achieve the best representation of O_2 concentrations at depth. The parameters b and c were chosen based on the best fit. The effective consumption rate R_{O2} in the model follows monod kinetics using equation 5, where Ks is the half-velocity constant [11]. Longitudinal and vertical transverse dispersivities were set to 0.05m and 0.005 m, respectively, in accordance with the investigated spatial scale of ~1m considered in this study. [12]

$$R_{O2} = R_{O2,\,max} \cdot \frac{c_{O2}}{(K_s + c_{O2})} \tag{S6}$$

The simulations were performed over 75 days for summer and 125 days for winter to ensure a quasi-steady-state distribution of O_2 during a tidal cycle.

Table S 2. Seasonal values for parameters used in the reaction model

Reaction Model Parameter	Summer	Winter
$O_{2,\ airsat}$	225 µmol l^{-1}	310 µmol l^{-1}
Aeration rate	0.1 $min^{-1} \cdot (O_{2,\ airsat} - O_2)$	0.1 $min^{-1} \cdot (O_{2,\ airsat} - O_2)$
a (equation S4)	0.19	0.1
b (equation S4)	1.22	3.67
c (equation S4)	0.17	0.84
K_s (equation S5)	10 µmol l^{-1}	10 µmol l^{-1}

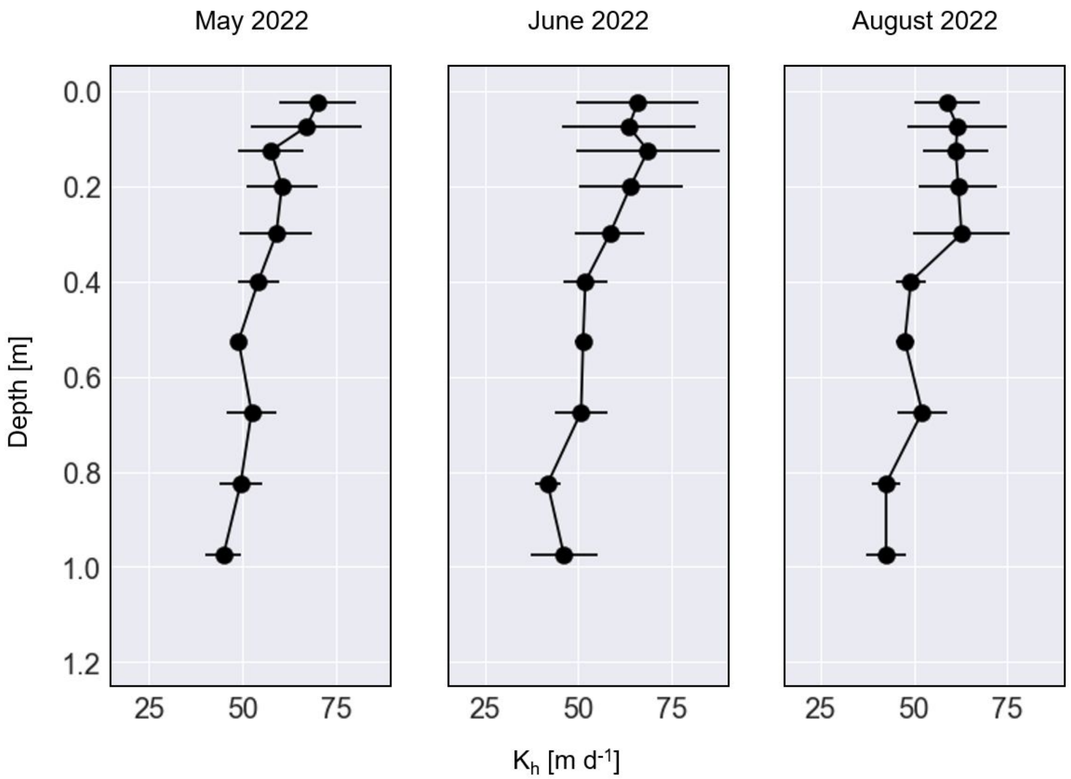

Figure S 1. Hydraulic conductivity estimates in the upper meter based on grain size d10 of the beach sediments for investigated campaigns (May - August 2022)

2. Continuous Oxygen Measurements

In December 2022, continuous in-situ O_2 measurements were conducted using sensor probes (Pyroscience OXROB) buried within the sediment at IT 2 and 3 in 50 cm depth for several tidal cycles. The probes were buried on 05th of December around 5 pm at low tide. The measurements show very weak fluctuations of O_2 levels (about 2.5 % airsat.) during tidal cycles. These principal O_2 dynamics during the tidal cycle align well with the modeled dynamics. However, the absolute O_2 concentrations as measured by the probes in the field are substantially lower than predicted by the model.

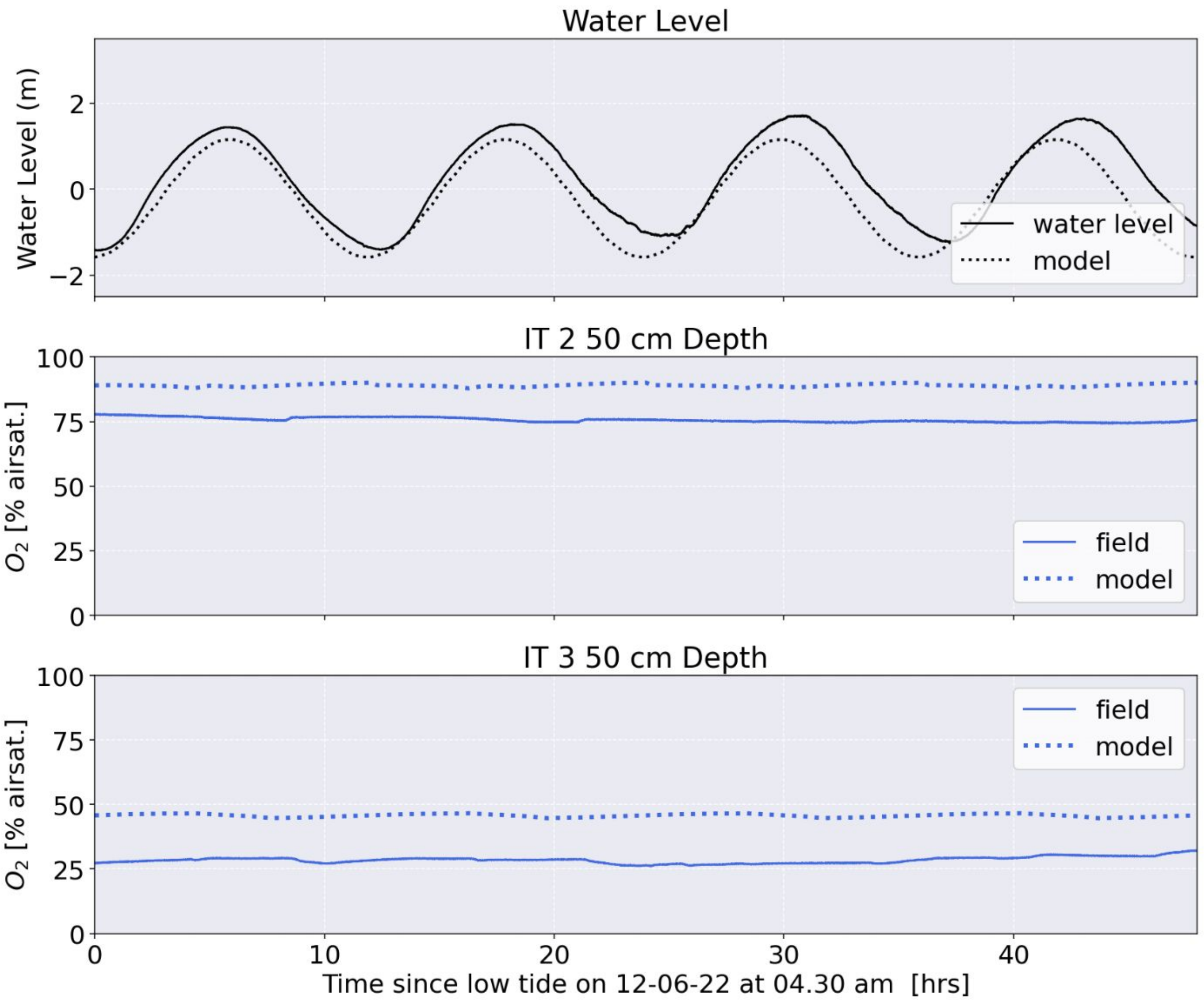

Figure S 2. Water level (upper panel) and O_2 variations (lower panels) during 4 tidal cycles in December 2022 as measured by buried sensor probes (solid line) and model (dotted line). The apparent delay in modeled water levels after the first tidal cycle arises from the implementation of a fixed 24%h tidal period, whereas actual tides follow a 24%h%50%min lunar day cycle. For oxygen, the buried probes capture the variability and trends over tidal cycles reliably but may show an offset in absolute values compared to modeled concentrations. This offset can arise from sediment disturbance during installation and limited porewater exchange at the sensor–sediment interface. The model, in turn, assumes rapid re-aeration of desaturated porewater in the upper sediment, which may oversimplify the dynamics in this highly reactive layer. Together, these factors explain the differences in absolute values but do not affect the conclusion that tidal-scale O_2 variability is relatively minor, consistent with model behavior. Water level measurements were provided by BSH. [6]

Supporting References

1. Nielsen, P. *Coastal and Estuarine Processes.* **29,** (WORLD SCIENTIFIC, 2009).
2. COMSOL®. Subsurface Flow Module Users Guide. (2024). at <https://doc.comsol.com/6.2/doc/com.comsol.help.ssf/SubsurfaceFlowModuleUsersGuide.pdf (accessed 2024-08-28)>
3. Van Genuchten, M. Th. A Closed-form Equation for Predicting the Hydraulic Conductivity of Unsaturated Soils. *Soil Science Soc of Amer J* **44,** 892–898 (1980).
4. BGR. Parameter für das Modell einer stetigen Funktion der θ (ψ)-Beziehung. (1999). at <https://www.bgr.bund.de/DE/Themen/Boden/Netzwerke/AGBoden/Downloads/Ergaenzungsregel_1_18.html (accessed 2024-10-09)>
5. Grünenbaum, N., Greskowiak, J., Sültenfuß, J. & Massmann, G. Groundwater flow and residence times below a meso-tidal high-energy beach: A model-based analyses of salinity patterns and 3H-3He groundwater ages. *Journal of Hydrology* **587,** 124948 (2020).
6. Bundesamt für Seeschifffahrt und Hydrographie (BSH) [Federal Maritime and Hydro-graphic Agency]. Wellenradar Nordergründe. (2023). at <https://www.bsh.de/DE/DATEN/Klima-und-Meer/Meeresumweltmessnetz/_Module/Info_Stationen/info_Nordergruende_Wellenradar_node.html (accessed 2023-11-13)>

7. Hölting, B. & Coldewey, W. G. *Hydrogeology.* (Springer Berlin Heidelberg, 2019). doi:10.1007/978-3-662-56375-5

8. Beck, M., Reckhardt, A., Amelsberg, J., Bartholomä, A., Brumsack, H.-J., Cypionka, H., Dittmar, T., Engelen, B., Greskowiak, J., Hillebrand, H., Holtappels, M., Neuholz, R., Köster, J., Kuypers, M. M. M., Massmann, G., Meier, D., Niggemann, J., Paffrath, R., Pahnke, K., Rovo, S., Striebel, M., Vandieken, V., Wehrmann, A. & Zielinski, O. The drivers of biogeochemistry in beach ecosystems: A cross-shore transect from the dunes to the low-water line. *Marine Chemistry* **190,** 35–50 (2017).

9. Auer, F., Ahmerkamp, S., Cueto, J., Winter, C. & Holtappels, M. Oxygen consumption rate, organic carbon and grain size data for intertidal sediments and oxygen concentration of pore waters data of Spiekeroog Island North Beach, May 2022 to April 2023. (2024). doi:https://doi.pangaea.de/10.1594/PANGAEA.971357

10. Reckhardt, A., Meyer, R., Seibert, S. L., Greskowiak, J., Roberts, M., Brick, S., Abarike, G., Amoako, K., Waska, H., Schwalfenberg, K., Schmiedinger, I., Wurl, O., Böttcher, M. E., Massmann, G. & Pahnke, K. Spatial and temporal dynamics of groundwater biogeochemistry in the deep subsurface of a high-energy beach. *Marine Chemistry* **264,** 104461 (2024).

11. Monod, J. THE GROWTH OF BACTERIAL CULTURES. *Annu. Rev. Microbiol.* **3,** 371–394 (1949).

12. Gelhar, L. W., Welty, C. & Rehfeldt, K. R. A critical review of data on field-scale dispersion in aquifers. *Water Resources Research* **28,** 1955–1974 (1992).
